# Second victim: after all, what is this?

**DOI:** 10.31744/einstein_journal/2020ED5619

**Published:** 2020-05-07

**Authors:** Alexsandro Tartaglia, Marcos Antonio Almeida Matos

**Affiliations:** 1 Escola Bahiana de Medicina e Saúde Pública SalvadorBA Brazil Escola Bahiana de Medicina e Saúde Pública, Salvador, BA, Brazil.

Healthcare professionals practice their activities daily with dedication and informed by science, and face several situations of excessively complex care. Nobel, courageous, and full of compassion, they increasingly want to save more lives, be useful; with the objective of achieving better results in care, they bravely face long working hours with no sleep, and are known as professionals that dedicate themselves constantly in work as in studies. Additionally, they have a method of working that requires physical disposition and agility in order to evaluate, diagnose, and care for their patients. Watching over the lives of others is the task of healthcare professionals.^(1,2)^

One of the major objectives of all professionals is to avoid complications, focused on safety of patients and quality of care. Nevertheless, adverse events are a reality and likely with always be a part of the system, due to the universal nature of human fallibility and the complexity in which care is inserted. Adverse events can cause severe damage to the patient or death; thus, the patient is the “first victim” of these events. However, the victims of adverse events go way beyond a given individual. When they occur, there is an indirect effect on healthcare professionals, who are considered the “second victims”.^(2)^

The term “second victim” was used by Albert Wu, professor of health policies and management at the Johns Hopkins Bloomberg School of Public Health, in the year 2000, to describe the impact of adverse events on healthcare professionals. Some symptoms experienced by them have been described in literature and include psychological (shame, guilt, anxiety, sadness, and depression), and cognitive (dissatisfaction, exhaustion, secondary traumatic stress) symptoms, besides physical reactions, with a negative impact on their bodies.^(2)^

It is probable that healthcare professionals directly involved in adverse events suffer emotional response reactions, which can lead to difficulty sleeping, guilt, lack of confidence, shame, anxiety, or reduced job satisfaction. If not treated, they may result in several consequences, including depression, emotional exhaustion, post-traumatic stress disorder, and suicidal ideation, according to a survey carried out by the American Society of Healthcare Risk Management, in 2015.^(1-4)^

An analysis of 2013 in reference to healthcare professional as “second victims”, and published in the Evaluation & the Health Professions, concluded that almost half of them experienced some impact, but few sought help.^(2,3)^

A study conducted in 2017, based on professional interviews linked to the patient safety sector in intensive care hospitals in Maryland, United States, highlighted numerous barriers impeding physicians, nurses, and other healthcare professional from receiving help after the occurrence of adverse events. The primary barriers included fear of confidentiality and the negative judgment of their work colleagues.^(3)^ Support programs to the “second victims” should be implemented, seeking to break these barriers and aggregate all healthcare professionals, giving them the aid they need.^(2,3)^

A survey performed with 898 physicians at the University of Missouri, also in the United States, found that the physicians, after the occurrence of an adverse event, wanted a support system that could relieve them from immediate patient care tasks for a brief time. Additionally, they desired to receive individual support and constant feedback, as well as access to specialists in patient safety and risk management, offering support and referrals to other specialists, when necessary. The healthcare system of the University of Missouri developed a support program implemented by a multiprofessional rapid response team.^(3)^The Johns Hopkins Hospital has a multidisciplinary work group dedicated to “second victims”, which develops its activities by helping the hospital provide care and support to the healthcare professionals involved in adverse events.^(3)^

Unfortunately, in Brazil, support programs for this situation are unknown, studied or valued very little within the area of patient safety, perpetuating and validating a punitive culture in the management of organizations.^(5)^A glimpse of this punitive, toxic, and individual approach culture of error was made evident in Brazilian nursing, by means of a documental investigation, from 1995 to 2010, with data collected from 13 ethical-disciplinary processes received by the Regional Council of Nursing of the State of Bahia (COREN-BA). In these processes, the health organizations - represented by the nursing coordination - stood out as whistleblowers of adverse events committed by nursing professionals.^(6)^ This sample, in fact, still represents a major setback in human resource management in health.

It is evident that there is a risk of human error behind each undertaking/action, but each person should be responsible only for activities under their control. For the safety of the patient, healthcare professionals should have appropriate tools and environment to carry out the tasks necessary and coordinate efforts.^(2)^ To blame only healthcare professionals is a commonplace approach in our midst, which represents an easier act than directing it, also, to organizations.^(2-6)^ Unfortunately, our culture has failed in the support to healthcare professionals involved in these cases. They declare that many organization reactions to adverse events are malicious, threatening, isolating, and fundamentally useless.^(2)^

Studies on adverse events affecting the patient described the main causes as lack of structural conditions in the work environment, inadequate materials and equipment, insufficient staff sizing, work overload, professional fatigue and stress, error in planning activities, process failures, and lack of effective communication.^(5,7)^

Recognizing the seriousness of this problem and its impact on patient care, the Joint Commission International (JCI) issued a statement, in January 2018, aimed to help healthcare organizations with recommendations and resources on how to support “second victims”. If not properly conducted, a “second victim” experience can bring physical and emotional harm to healthcare professionals who work both to treat and care for patients. Several of these victims, however, need support and care that many health organizations are not prepared to offer. This underscores the importance of establishing “second victim” programs, which play a critical role in strengthening the culture of safety and reducing stigma and prejudice. The guide briefly provides safety actions for health organizations to consider, including developing a culture for learning from health system adversities and communicating lessons learned, as well as guidance on how staff can support themselves when adverse events occur.^(2)^ Leadership resistance is among the most significant barriers to creating an effective culture to support “second victims”. This is usually because the value and purpose of these targeted programs have not not been clearly understood.^(2)^ The role of leaders is extremely important, since they provide empathy and emotional support to the professional. The best strategy seems to be creating support networks at individual, organizational, or national levels. When initiating a significant accusation of adverse event, in addition to investigating the root cause, a parallel analysis should be made to determine if there are “second victims”.^(3)^

A change of culture is needed in health care, with a transfer of the traditional one, in which shame, guilt and punishment directed at health professionals who have experienced a “second victim” phenomenon, must be quickly replaced by a movement towards a just culture, in which each one takes responsibility for the activities under their control.^(3)^

It is crucial that patients and families affected by adverse events receive more attention. On the other hand, care and attention should also be given to “second victims”.^(3)^

We emphasize the need to further recognize the nature of the “second victim” phenomenon and establish organizational support for affected healthcare professionals. An organizational environment must be built in which these sensitive issues are discussed in a clear, non-judgmental and non-punitive manner. We also emphasize the need for well-established support structures, which can meet the needs of the healthcare professionals involved.
