# Supplementary material for: Second victim: after all, what is this?
Source: Einstein (Sao Paulo). 2020 May 7;18:eED5619. doi: 10.31744/einstein_journal/2020ED5619 (PMC7206981; doi:10.31744/einstein_journal/2020ED5619)
Supplement: Translation

# Translation: Segunda vítima: afinal, o que é isso?

**Authors:** Alexsandro Tartaglia, Marcos Antonio Almeida Matos

**Affiliations:** 1 Brasil Escola Bahiana de Medicina e Saúde Pública, Salvador, BA, Brasil.

Os profissionais de saúde praticam suas atividades diariamente, com ciência e dedicação, em diversas situações de cuidados excessivamente complexos. Nobres, corajosos e cheios de compaixão, querem cada vez mais salvar pessoas, serem úteis, objetivam melhores resultados na assistência, enfrentam bravamente longas jornadas de trabalho e horas sem dormir, e são conhecidos como profissionais que se dedicam constantemente, tanto no trabalho quanto nos estudos. Além disso, possuem uma forma de trabalhar que requer disposição física e agilidade para avaliar, diagnosticar e cuidar de seus pacientes. Zelar pela vida dos outros é tarefa dos profissionais de saúde.^(1,2)^

Evitar complicações é um dos principais objetivos de todos os profissionais, com foco na segurança do paciente e na qualidade da assistência. No entanto, eventos adversos são uma realidade e, provavelmente, sempre farão parte do sistema, devido à natureza universal da falibilidade humana e à complexidade na qual os cuidados se inserem. Os eventos adversos podem causar danos graves ao paciente ou óbito; assim, o paciente é a “primeira vítima” desses eventos. No entanto, as vítimas de eventos adversos vão muito além de tal indivíduo. Quando eles ocorrem, há um efeito indireto nos profissionais de saúde, considerados as “segundas vítimas”.^(2)^

O termo “segunda vítima” foi usado, em 2000, por Albert Wu, professor de política e gestão em saúde na *Johns Hopkins Bloomberg School of Public Health*, para descrever o impacto dos eventos adversos nos profissionais de saúde. Alguns sintomas vividos por eles foram descritos na literatura e incluem manifestações de cunhos psicológico (vergonha, culpa, ansiedade, tristeza e depressão) e cognitivo (insatisfação, desgaste e estresse traumático secundário), além de reações físicas, com impacto negativo em seu organismo.^(2)^

É provável que profissionais de saúde diretamente envolvidos em eventos adversos sofram reações de resposta emocional, que podem levar à dificuldade para dormir, culpa, insegurança, vergonha, ansiedade ou redução da satisfação no trabalho. Se não tratados, podem resultar em algumas consequências, incluindo depressão, desgaste, transtorno de estresse pós-traumático e ideação suicida, de acordo com pesquisa realizada pela *American Society of Healthcare Risk Management*, em 2015.^(1-4)^

Uma análise de 2013 referente aos profissionais de saúde como “segundas vítimas”, publicada na *Evaluation & the Health Professions*, concluiu que quase metade deles vivencia algum impacto, mas poucos procuram ajuda.^(2,3)^

Estudo de 2017, baseado em entrevistas profissionais ligadas ao setor de segurança do paciente em hospitais de cuidados intensivos em Maryland, nos Estados Unidos, destacou inúmeras barreiras impedindo que médicos, enfermeiros e outros profissionais de saúde obtivessem ajuda após a ocorrência de eventos adversos. As principais barreiras incluíam o medo de confidencialidade e o julgamento negativo dos colegas de trabalho.^(3)^ Programas de apoio às “segundas vítimas” devem ser implementados, buscando romper essas barreiras e agregar todos os profissionais de saúde, dispensando o apoio de que precisam.^(2,3)^

Pesquisa realizada com 898 médicos clínicos da *University of Missouri*, também nos Estados Unidos, constatou que os médicos, após a ocorrência de um evento adverso, desejavam um sistema de suporte que pudesse aliviá-los das tarefas imediatas de atendimento ao paciente por um breve período. Além disso, gostariam de receber suporte individual e *feedback* constante, bem como acesso a especialistas em segurança do paciente e gerenciamento de risco, oferecendo apoio e encaminhamento para outros especialistas, quando necessário. O sistema de saúde da *University of Missouri* desenvolveu um programa de suporte implantado por uma equipe interprofissional de resposta rápida.^(3)^ O *Johns Hopkins Hospital* possui um grupo de trabalho multidisciplinar dedicado às “segundas vítimas”, o qual desenvolve suas atividades auxiliando o hospital a prestar cuidados e apoio aos profissionais de saúde envolvidos em eventos adversos.^(3)^

Infelizmente, no Brasil, desconhecem-se programas de apoio a essa situação, pouco estudada ou valorizada dentro da área de segurança do paciente, perpetuando e validando uma cultura punitiva na gestão das instituições.^(5)^ Um recorte dessa cultura punitiva, tóxica e de abordagem individual do erro foi evidenciado na enfermagem brasileira, por meio de pesquisa documental, de 1995 a 2010, com dados coletados a partir de 13 processos ético-disciplinares recebidos pelo Conselho Regional de Enfermagem da Bahia (COREN-BA). Nestes, as organizações de saúde, representadas pela coordenação de enfermagem, destacaram-se como denunciantes dos eventos adversos cometidos por profissionais de enfermagem.^(6)^ Essa amostra, de fato, ainda representa um grande retrocesso na gestão de recursos humanos em saúde.

É evidente que existe um risco de erro humano por trás de cada empreendimento/ação, mas cada pessoa deve ser responsabilizada apenas por atividades sob seu controle. Para a segurança do paciente, os profissionais de saúde devem ter ferramentas e ambiente adequados para executar as tarefas necessárias e coordenar esforços.^(2)^ Culpar somente os profissionais de saúde é uma abordagem corriqueira em nosso meio, o que representa um ato mais fácil do que direcioná-la, também, às instituições.^(2-6)^ Infelizmente, nossa cultura tem faltado no apoio aos profissionais de saúde envolvidos nesses casos. Eles declararam que muitas reações organizacionais a eventos adversos são maliciosas, ameaçadoras, isolantes e fundamentalmente inúteis.^(2)^

Estudos sobre eventos adversos que atingem o paciente descrevem as principais causas falta de condições estruturais no ambiente de trabalho, materiais e equipamentos inadequados, dimensionamento insuficiente de pessoal, sobrecarga de trabalho, cansaço e estresse do profissional, erro de planejamento das atividades, falhas de processo e ausência de comunicação efetiva.^(5,7)^

Reconhecendo a gravidade desse problema e seu impacto no atendimento ao paciente, a *Joint Commission International* (JCI) emitiu um comunicado em janeiro de 2018 destinado a ajudar as organizações de saúde, com recomendações e recursos sobre como apoiar as “segundas vítimas”. Uma experiência de “segunda vítima”, se não conduzida adequadamente, pode trazer danos físicos e emocionais aos profissionais de saúde, que trabalham tanto para tratar como cuidar dos pacientes. Várias dessas vítimas, no entanto, precisam de apoio e cuidados que muitas organizações de saúde não estão preparadas para oferecer. Isso enfatiza a importância do estabelecimento de programas de “segundas vítimas”, que desempenham papel crítico no fortalecimento da cultura de segurança, além de reduzirem estigmas e preconceitos. O guia oferece, resumidamente, ações de segurança a serem consideradas pelas organizações de saúde, incluindo o desenvolvimento de uma cultura para aprender com as adversidades do sistema de saúde e a comunicação das lições aprendidas, além de orientações sobre como a equipe pode se apoiar na ocorrência de um eventos adversos.^(2)^

A resistência da liderança está entre as barreiras mais significativas à criação de uma cultura eficaz em torno do apoio às “segundas vítimas”. Isso geralmente ocorre porque o valor e o propósito desses programas direcionados não são claramente compreendidos.^(2)^ O papel dos líderes é extremamente importante, pois eles fornecem empatia e apoio emocional ao profissional. A melhor estratégia parece ser a criação de redes de suporte em níveis individual, organizacional ou nacional. Ao iniciar uma acusação de eventos adversos significativo, além da investigação da causa raiz, deve ser iniciada uma análise paralela para determinar se existem “segundas vítimas”.^(3)^

É necessária uma mudança de cultura na assistência à saúde, com transferência da tradicional, em que a vergonha, a culpa e a punição voltadas para os profissionais de saúde que experimentaram um fenômeno de “segundas vítimas” devem ser rapidamente substituídas por um movimento em direção a uma cultura justa, na qual cada um se responsabilize pelas atividades sob seu controle.^(3)^

É crucial que pacientes e famílias prejudicados por eventos adversos recebam mais atenção. Por outro lado, devem também ser dispensados cuidados e atenção às “segundas vítimas”.^(3)^

Ressaltamos a necessidade de reconhecer ainda mais a natureza do fenômeno da “segundas vítima” e estabelecer apoio organizacional para os profissionais de saúde afetados. Deve ser construído um clima organizacional em que essas questões delicadas sejam discutidas de maneira clara, não julgadora e não punitiva. Enfatizamos, ainda, a necessidade de estruturas de suporte bem estabelecidas, que possam atender às necessidades dos profissionais de saúde envolvidos.
